# Use of and Experiences With Online Access to Electronic Health Records for Parents, Children, and Adolescents: Protocol for a Scoping Review

**DOI:** 10.2196/36158

**Published:** 2022-06-15

**Authors:** Josefin Hagström, Charlotte Blease, Barbara Haage, Isabella Scandurra, Scharlett Hansson, Maria Hägglund

**Affiliations:** 1 Department of Women's and Children's Health Uppsala University Uppsala Sweden; 2 Beth Israel Deaconess Medical Center Harvard Medical School Boston, MA United States; 3 Department of Health Technologies Tallinn University of Technology Tallinn Estonia; 4 Informatics Örebro University School of Business Örebro Sweden

**Keywords:** electronic health record, patient-accessible electronic health record, adolescents, parents, children, patient experience, patient portal, electronic portal, review, scoping review, youth, patient perspective, user experience, patient access

## Abstract

**Background:**

As patient online access to electronic health records becomes the standard, implementation of access for adolescents and parents varies across providers, regions, and countries. There is currently no international compilation of evidence to guide policy decisions in matters such as age limit for access and the extent of parent proxy access.

**Objective:**

This paper presents the protocol for a scoping review of different stakeholders’ (including but not limited to end users) perspectives on use, opinions, and experiences pertaining to online access to electronic health records by parents, children, and adolescents.

**Methods:**

This scoping review will be conducted according to the Arksey and O’Malley framework. Several databases will be used to conduct a literature search (PubMed, CINAHL, and PsycInfo), in addition to literature found outside of these databases. All authors will participate in screening identified papers, following the research question: How do different stakeholders experience parents’, children’s, and adolescents’ online access to the electronic health records of children and adolescents? Data abstraction will include but will not be limited to publication type, publication year, country, sample characteristics, setting, study aim, research question, and conclusions. The data to be analyzed are from publicly available secondary sources, so this study does not require an ethics review.

**Results:**

The results from this scoping review will be presented in a narrative form, and additional data on study characteristics will be presented in diagrams or tabular format. This scoping review protocol was first initiated by Uppsala University in June 2021 as part of the NordForsk-funded research project NORDeHEALTH. The results are expected to be presented in a scoping review in June 2022. The results will be disseminated through stakeholder meetings, scientific conference presentations, oral presentations to the public, and publication in a peer-reviewed journal.

**Conclusions:**

This is, to our knowledge, the first study to map the literature on the use and experiences of parents’ and adolescents’ online access to the electronic health records of children and adolescents. The findings will describe what benefits and risks have been experienced by different stakeholders so far in different countries. A mapping of studies could inform the design and implementation of future regulations around access to patient-accessible electronic health records.

**International Registered Report Identifier (IRRID):**

DERR1-10.2196/36158

## Introduction

### Background

Digitalized health records, also called electronic health records (EHRs), contain clinical information (eg, doctor visit notes, lists of medications, and diagnostic information) and are used by health care professionals. Technological advancements have enabled patients to read their EHRs via online patient portals, often called patient-accessible EHRs (PAEHRs), quickly and easily, which promotes patient empowerment. It appears that PAEHRs are becoming the standard [1-5], as an increasing number of patients worldwide gain access to their records [6]. Today, health institutions in over 15 countries are providing patients with online access to their medical records via secure online portals [4]. Furthermore, a recent US policy of “open notes” mandates health care providers by law to share the records with patients [7]. In response to this rapid development, legal frameworks are continuously being adapted to improve use and ensure privacy of such PAEHR systems [4,8].

As PAEHRs continue to be implemented worldwide, vast uncertainty remains in the area of access by parents, children, and adolescents [9]. This is evident from the variation in the age of a child at which parents gain and lose access as well as the age at which young patients can access their records on their own [4]. On a national level, some countries (eg, Sweden and Finland) hold nationally regulated systems while others use a case-by-case approach (eg, the United States and New Zealand) [4]. In some countries, parents and guardians (herein, referred to as parents) are offered access while in other countries, parents are blocked from accessing records by law when their children reach a certain age threshold. In Sweden, for example, a parent has default access to their child’s PAEHR until the child turns 13 years old, and the age limit for accessing one’s own data is 16 years. Thus, no one has access to the child’s EHR when the child is between 13 and 15 years of age. At this point, adolescents in Sweden can decide to provide their parent(s) with continued access to their records through an administrative process requiring approval by a health care professional. In Australia, on the other hand, adolescents can make similar decisions with a click on their computer. In France, adolescents receive access at 18 years of age when, in turn, the parent loses access. Decisions about earlier access in France may also depend on the perceived maturity of the minor. In many countries and regions, a lack of user continuity of access is apparent [4]. Currently, there is no international consensus on PAEHR regulations for parents, children, and adolescents.

For the most part, PAEHRs have been investigated for the general adult population. Effects of PAEHRs are not conclusive, yet indicate benefits including improved medication adherence and self-care, as well as improved relationships between patients and their physicians [3,10,11]. However, a growing body of literature is exploring access to PAEHRs for parents, children, and adolescents in particular. Patient online access to EHRs during the transition from child to adult is complex; parental access, while often appreciated by parents [12], may lead to ethical challenges. For example, some health information may be considered sensitive by adolescents, such as health care data pertaining to the disclosure of alcohol or drug abuse, sexual activity, or stigmatized illnesses such as anxiety or depression. Adolescents have also been observed to withhold information from health care professionals if they are uncertain about who may access it [13,14]. Furthermore, it has been suggested that adolescents’ acceptability of parental PAEHR access will vary depending on the relationship with their parent(s) [15]. With regard to adolescents’ own access, a strong desire for control has been expressed [16] while health care professionals have expressed concerns [17]. Therefore, while it appears that PAEHR access offers information transparency that might contribute to patient empowerment and enhanced health care [3,18], evidence suggests the adolescent population requires targeted analysis.

### Study Objectives

The objective of the proposed scoping review is to identify, categorize, and summarize knowledge about different stakeholders’ (eg, children and adolescents, parents, health care professionals, policy-makers, and designers of patient portals or PAEHRs) use and experiences of PAEHR access for parents, children, and adolescents. Countries are currently at different stages of development and implementation of PAEHRs; therefore, compiling the literature is timely and has, to our knowledge, not yet been undertaken. This scoping review is anticipated to aid policy-makers in designing future regulations around PAEHR access for parents and adolescents, and to potentially improve the design and implementation of PAEHRs to meet the needs of end users.

## Methods

### Approach

A scoping review will be conducted using the Arksey and O’Malley [19] framework. The framework includes 6 stages: (1) identifying the research question; (2) identifying relevant studies; (3) study selection; (4) charting the data; (5) collating, summarizing, and reporting the results, and (6) consulting with relevant stakeholders. Methodological comments on the framework will be consulted during the process to enhance the method [20-22].

#### Stage 1: Identifying the Research Question

Through discussion among research team members, the main research question is: How do different stakeholders experience parents’, children’s, and adolescents’ online access to the electronic health records of children and adolescents? We do not limit the question only to children’s and adolescents’ or parents’ experiences but also include other relevant stakeholders including health care professionals and policy-makers. For this review, PAEHR access is defined as access provided via an online patient portal that can encompass the entire electronic record or parts of it (eg, access to test results, clinical notes, or medications). The practice of “open notes” is included in the concept of EHR access [1,23], referring specifically to health care professionals sharing the visit note summaries they write with patients.

#### Stage 2: Identifying Relevant Studies

**Figure 1:**
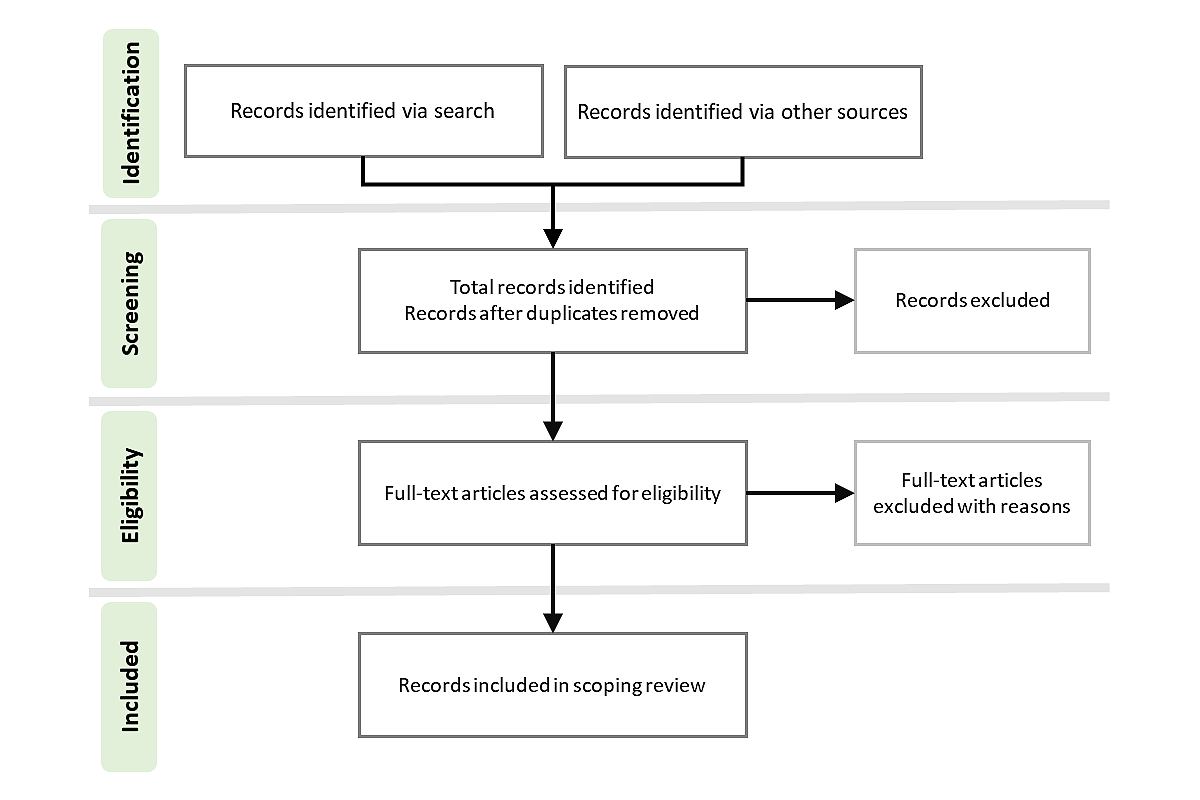
The search strategy for the scoping review.

The literature search will be carried out by an experienced research librarian at Uppsala University. The search strategy, presented in Figure 1, is designed to include formally published peer-reviewed articles and selected gray literature (eg, dissertations, conference abstracts, editorials, and letters). Published works will be identified using the following electronic literature databases: PubMed, CINAHL, and PsycInfo. The time frame for the search will be 2005 onwards. Search terms will be identified with input from the research team and the literature. The search term is based on 3 key concepts: (1) EHR, (2) sharing EHRs with service users, and (3) pediatric or adolescent access, which will be combined with the Boolean operator “AND.” The following search string will be used and adapted for the different databases: (“open notes” OR “opennotes” OR ((“health record” OR “patient record” OR “pediatric record” OR “clinical record” OR “health notes” OR “clinical notes” OR “pediatric notes”) AND (access OR show OR open OR share OR read OR participant*)) AND (pediatric OR adolescent* OR parent*)). Subsequently, references in the retrieved articles will be scanned backward to identify prior work that should be considered for the research topic. The key concept “PAEHR” is considered redundant, as it is covered in the “AND access” search term. Furthermore, the authors will be able to include records found but not identified in the search.

#### Stage 3: Selection of Eligible Studies

The scientific literature will be systematically compiled and the selection will be inclusive, striving to encompass publications and reports that employ a variety of methodologies. Inclusion and exclusion criteria are informed by the review process and will be applied at the study selection stage.

##### Inclusion Criteria

Studies will be included if they meet the following criteria:

- Patient user population: parents, children, and adolescents
- Population studied: parents, children, adolescents, and health care professionals
- Outcomes: use, implementation, and experiences of access or proxy access to PAEHRs
- Study design: all study types

##### Exclusion Criteria

Studies will be excluded if they:

- Are not written in English
- Were published outside the study period
- Do not focus on PAEHRs

##### Search Strategy

The research team will identify eligibility criteria and search terms. A software program, Rayyan [24], will be used during the screening process after which included articles will be extracted into an Excel spreadsheet (Microsoft Corp) to facilitate analysis. The first author will set up the Excel spreadsheet and have the main responsibility of verifying the accuracy of its data. Study titles and abstracts will be independently screened by 5 investigators. Next, full-text articles will be divided among the 5 investigators so that each article is screened by at least 2 people. Where disagreements arise, these will be resolved by a third reader, and, if necessary, by group discussion.

#### Stage 4: Data Collection

Study characteristics will be identified by the research team and extracted into the Excel spreadsheet created in stage 3. Characteristics will include but will not be limited to publication type, publication year, country, sample characteristics, setting, study aim, research question, and conclusions. All researchers will be able to contribute to the spreadsheet. Ideas emerging during the process will be discussed among the authors in regular meetings set up by the main author.

#### Stage 5: Data Summary and Synthesis of Results

Results reported in the included studies will be compiled and read multiple times. Results will then be analyzed independently by 2 researchers (JH and MH) using thematic analysis [25]. In this process, the analytical material will be further summarized, and key themes will be identified to organize the study results. The results of this synthesis process will be discussed and approved by the entire research team. Tentative themes include but are not limited to positive and negative experiences, concerns, and benefits, as informed by a previous scoping study in a similar area [26].

#### Stage 6: Consultation

Because consultation can provide additional information and insights [21], the results of the literature review will be presented to and discussed with important stakeholder representatives from pediatric care, including a pediatric oncologist, a young patient council at a public hospital in Sweden, and the Ombudsman for Children in Sweden. These stakeholder representatives will be provided with material via email. The youth panel will discuss these results in a meeting, and all 3 stakeholder representatives will be able to choose whether to provide their thoughts in text via email or verbally in a Zoom (Zoom Video Communications, Inc) meeting.

### Ethical Considerations

As the scoping review methodology consists of reviewing publicly available materials only, this study is not subject to ethical approval.

## Results

The main results of our analysis will be presented in a narrative form focusing on research results to date regarding different stakeholders’ experiences of providing children and adolescents and their parents with online access to their EHRs. Additional data on year, country, study design, study population, and setting will be presented in diagrams or tabular format. This scoping review protocol was first initiated by Uppsala University in June 2021 as part of the NordForsk-funded research project NORDeHEALTH. We expect the results to be presented in a scoping review in June 2022.

## Discussion

The results from this scoping review will aim to inform a variety of stakeholders, including policy- and decision-makers, vendors, designers of patient portals and PAEHRs, and perhaps most importantly, end-user representatives. We aim to describe the benefits and risks experienced by different stakeholders so far in different countries. This knowledge may improve both the design and implementation of future PAEHRs to become more useful to the population, and also guide policy-makers and other decision-makers to provide the right preconditions for future implementations. In both Sweden and Estonia, the current patient portals are being redesigned, and there may be opportunities to influence both portal design and policy development. Therefore, results will be communicated outside the traditional scientific publications, through, for example, seminars and reports focusing specifically on the context in Sweden and Estonia. Results that are of interest to parents, adolescents, and health care professionals (eg, reports on the benefits or risks of record access) will be shared in more easily accessible formats like social media communications, popular science publications, and presentations for practitioners. We hope that this may have a direct impact on how record access is used by health care professionals, parents, and adolescents to increase potential benefits and minimize any risks.

To date, several literature reviews have been performed regarding PAEHRs or open notes in general [10,27,28], but to our knowledge, this will be the first review focusing specifically on the unique challenges in this particular subgroup. We also aim to identify current knowledge gaps in parents’ and children’s access to EHRs to guide future research in this area.

## Abbreviations

EHR: electronic health record
PAEHR: patient-accessible electronic health record
